# Significant change of spin transport property in Cu/Nb bilayer due to superconducting transition

**DOI:** 10.1038/srep06260

**Published:** 2014-09-02

**Authors:** Kohei Ohnishi, Yuma Ono, Tatsuya Nomura, Takashi Kimura

**Affiliations:** 1 Department of Physics, Kyushu University, 6-10-1 Hakozaki, Fukuoka 812-8581, Japan; 2 Research Center for Quantum Nano-Spin Sciences, Kyushu University, 6-10-1 Hakozaki, Fukuoka 812-8581, Japan; 3 CREST, Japan Science and Technology Agency, Sanbancho, Tokyo 102-0075, Japan

## Abstract

The combination between the spin-dependent and super-conducting (SC) transports is expected to provide intriguing properties such as crossed Andreev reflection and spin-triplet superconductivity. This may be able to open a new avenue in the field of spintronics, namely superconducting spintronics because a superconductor itself has great potential for future nanoelectronic applications. To observe such SC spin transports, the suppression of the extrinsic effects originating from the heating and Oersted field due to the electric current is a crucial role. Pure spin current without accompanying the charge current is known as a powerful mean for preventing such extrinsic effects. However, non-negligible heat flow is found to exist even in a conventional pure spin current device based on laterally-configured spin valve because of the heating around the spin injector. Here, we develop a nanopillar-based lateral spin valve, which significantly reduces the heat generation, on a superconducting Nb film. By using this ideal platform, we found that the spin absorption is strongly suppressed by the SC transition of Nb. This demonstration is the clear evidence that the super-conducting Nb is an insulator for the pure spin current.

Manifestation of the conduction electrons in a solid state is one of the most important aspects for the electrical transports in the condensed matter physics. The strong correlation between the electron transport properties and the electron spin, namely spin-dependent transports, has received the considerable attentions after the discovery of giant magneto-resistance (GMR) effect^1^,^2^,^3^. Owing to the developments of the theoretical understanding and experimental techniques, the spin current, which is a flow of the spin angular momentum, is found to play the central role for the electron transports in ferromagnetic/nonmagnetic hybrid nanostructures. Especially, a pure spin current, which is a spin current without accompanying the charge current, enables the precise and sensitive detection of the spin-related phenomena such as spin Hall^4^,^5^,^6^ and spin Seebeck^7^,^8^ effects.

Spin currents, so far, have been investigated mainly in normal-conductor/ and semiconductor/ferromagnet hybrid systems intensively. The combination between the spin and super-conducting (SC) transports is also expected to provide intriguing transport properties, leading to an emerging field in spintronics because a superconductor itself has great potential for future nanoelectronic applications such as Josephson circuit and quantum computing^9^,^10^. Although a number of experimental studies have been performed^11^,^12^,^13^,^14^,^15^,^16^,^17^,^18^,^19^,^20^,^21^, several intriguing phenomena suggested in theoretical study have not been demonstrated^22^,^23^,^24^. Moreover, the experimental study on subgap transports such as a crossed Andreev reflection and a spin triplet proximity effect have still exciting topics in superconducting spintronics because of their fantastic properties^25^,^26^,^27^,^28^,^29^.

To investigate aforementioned phenomena in more detail, use of the pure spin currents yields several advantages compared with the spin-polarized current because the charge current induces spin-independent extrinsic effects such as the Joule heating and Oersted field^15^,^16^. The pure spin current is, in general, generated by nonlocal electrical spin injection using the laterally configured ferromagnetic/nonmagnetic hybrid structures^30^. However, even in such structures, the influence of the heat around the injecting junction significantly affects the superconducting properties. In fact, the bias current in the lateral spin valves is found to induce the strong suppression of the superconducting gap^21^. As a result, the spin polarized electron can enter the superconductor as quasi-particles. Moreover, the geometrical disorders in laterally configured multi-terminal devices may produce the extrinsic spin scattering. These deteriorations make it difficult to observe the drastic change of the spin transport by the superconducting transition. Therefore, an improved device structure is required for further developments of superconducting spintronics. We have recently developed an innovative device structure, nanopillar-based lateral spin valve, for generating the nonlocal spin current efficiently^31^,^32^. In this structure, the heat from the spin injector can be suppressed significantly because of the minimized channel length of the spin injector. In the present letter, we investigate the transport properties of the pure spin current in a nonmagnetic/superconducting interface by employing the nanopillar-based lateral spin valve structure.

Figure 1 shows a scanning electron microscope image of the fabricated device together with the schematic illustration of the device. First, we have prepared the Py/Cu/Nb trilayer film by using a high vacuum sputtering and evaporation system with the base pressure less than 2 × 10^−5^ Pa. Here, Nb and Py are 45-nm thick and 30-nm thick, respectively, and were deposited by the magnetron sputterings. The Cu spacer whose thickness is 100 nm was deposited by the Joule evaporation. Then, the trilayer film was patterned into the nanopillar structure by using electron-beam lithography and Ar ion milling. The detail of the fabrication procedure is described in our previous paper^31^. The lateral dimensions for two ferromagnetic nanopillars are 270 × 220 nm^2^ and 340 × 240 nm^2^, and the center-center distance between two ferromagnetic nanopillars is approximately 500 nm. Since the trilayer was prepared by in-situ multi-layered film grown system, the interfaces between each metal have ideal conditions.

To evaluate the transport property of the prepared film, we first measured the temperature dependence of the resistance for the Cu/Nb bilayer by using the probe configuration shown in Fig. 2(b). Here, the measurement was performed by the current-bias lock-in technique with the current amplitude of 180 *μ*A. As shown in Fig. 2(a), the resistance shows a typical normal conducting (NC) behavior with the residual resistance ratio of 3.2 above 7.1 K and becomes the zero-resistive superconducting state below 7.1 K. These characteristics indicate that the current flows mainly in the Cu film above 7.1 K and the current flows in the superconducting (SC) Nb film below 7.1 K, as schematically shown in Fig. 2(b). The transition temperature higher than the typical values in the patterned Nb thin films and the abrupt resistance change at 7.1 K indicate that the Nb layer has great performances as the superconductor^33^.

We then evaluated the spin transport by mean of the nonlocal spin valve measurement using the Py nanopillars. Here, the injection of the spin-polarized electrons from the Py nanopillar (Py1) into the Cu/Nb bilayer produces the spin current both in the left-hand and right-hand side. The pure spin current is produced in the right-hand side, and can be detected by measuring the electrical voltage using another Py nanopillar (Py2)^32^. The voltage difference between parallel and anti-parallel states divided by the injecting current is known as a spin signal. The detected spin signal reflects the magnitude of the spin current at the Py2/Cu interface. Before showing the results in the bilayer film, we show a typical nonlocal spin valve signal in the Py/Cu nanopillar lateral spin valve without Nb layer in Fig. 3(a), as a reference. A spin signal with the magnitude of 0.85 mΩ was clearly observed at 10 K. The spatial homogeneity of the spin injection using the Py nanopillar was evaluated by changing the current- and voltage-probe positions in the nonmagnetic channels. We confirmed that the obtained spin signals in two configurations are almost same. This indicates that the Py nanopillar produce the homogeneous spin accumulation underneath the injecting electrode. In the Cu/Nb bilayer film, since the spin relaxation process in the Cu channel is affected by the electron conducting state in the Nb, the spin signal should be modified from that in the reference sample^34^. In addition, the significant change should be observed in the spin signal by the transition from the NC to the SC in the Nb layer.

We then show the results for the spin transports in the Cu/Nb bilayer film. Figure 3(b) shows the nonlocal spin valve curve measured at 10 K with the current amplitude of 180 *μ*A. Although the clear resistance change between the parallel and anti-parallel state was observed, the obtained spin signal is 0.17 mΩ. This strong reduction of spin signal clearly indicates that the NC Nb layer strongly absorbs the spin current flowing in the Cu layer because of the strong spin relaxation of the Nb, as schematically shown in the left-hand side of Fig. 4^6^,^16^,^34^. The nonlocal spin valve curve measured at 2.3 K is shown in Fig. 3(c). Here, the bias current is also 180 *μ*A. The spin signal is enhanced by a factor of 5 with respect to that at 10 K, indicating that the spin absorption is strongly suppressed by the SC transition of the Nb layer. This can be understood as follows. At the NC Cu/SC Nb interface, in order to inject the electrons into the superconductor, the electrons have to be transformed into a Cooper pair consisting of two electrons with opposite spins^11^,^35^. However, the Cooper pairs cannot be formed from the pure spin current, in which the up-spin and down-spin electrons flow oppositely each other, as schematically shown in the right-hand side of Fig. 4. This is a clear demonstration that the SC gap suppresses the spin current. It also should be noted that the background resistance of the nonlocal signal at 2.3 K was almost zero while the large background around 40 mΩ was observed at 10 K. The large background at 10 K is caused by the spreading of the electric flux line originating from the quasi-two dimensional nonmagnetic Cu channel. But, it can be eliminated by the superconducting Nb channel. Thus, the SC Nb layer efficiently transforms the charge current into the super current. We also mention that the magnitude of the spin signals both at 2.3 K and 10 K was almost constant under the bias current below 300 μA. This is a strong evidence that the nanopillar structure developed here strongly reduces the heat generation^31^.

Finally, we focus on the spin transport in the Cu layer in which the Cooper pairs exist because of the proximity effect. Influence of the Cooper pair on the non-equilibrium spin accumulation was studied. Figure 5 shows the magnetic field dependence of the contact resistance for the Py1/Cu junction. The probe configuration for this measurement is shown in the inset. The small abrupt resistance changes in low magnetic field are due to the anisotropic magnetoresistance of the Py nanopillar. The gradual reversible resistance change below 100 mT can be understood by the field dependence of the resistance for the Cu layer. Owing to the proximity effect from the Nb, the current flowing around the Cu/Nb interface becomes the super current. As a result, the resistance of the Cu around the interface becomes zero in the absence of the magnetic field. However, the super current induced by the proximity effect is easily spoiled by applying the magnetic field. Thus, the gradual field dependence of the resistance is the evidence that Cooper pairs exist in the Cu channel. It should be noted that the field dependence of the nonlocal spin valve signal does not show such a gradual change. This indicates that the spin relaxation process in the Cu layer is not affected by the proximity effect from the superconductor.

In summary, we have developed an innovative device structure for investigating the transport of the pure spin currents in a superconductor/ferromagnet hybrid structure. We experimentally demonstrated that the pure spin current is effectively reflected by the superconducting Nb although the normal-conducting Nb effectively absorbs the pure spin current. Using the proximity effect, we were able to stabilize the Cooper pairs with the non-equilibrium spin accumulation in the Cu. We found that the spin relaxation process in the Cu layer is not affected by the Cooper pairs.

## Author Contributions

Y.O. and T.N. carried out the experimental work, including the preparation of the sample. K.O. carried out the analysis of the experimental results. K.O. and T.K. supervised the experimental research and wrote the main manuscript text including the figure preparations.

## Figures and Tables

**Figure 1:**
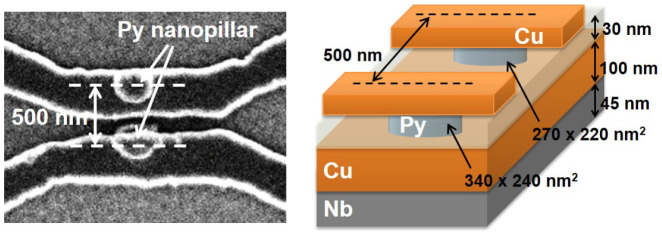
Scanning electron microscope image together with a schematic illustration of the prepared nano-pillar-based lateral spin valve. Two Py nanopillars are placed on the Cu/Nb bilayer film and are connected to the top Cu electrodes.

**Figure 2:**
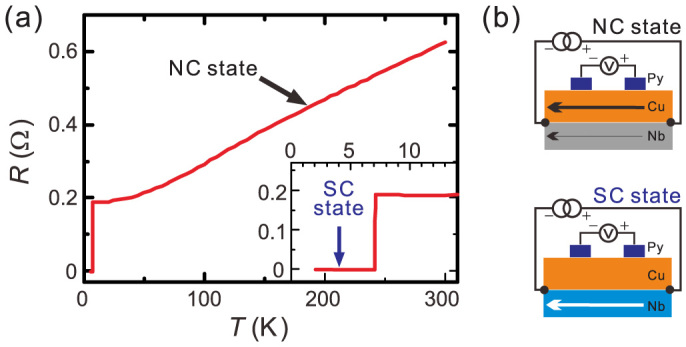
(a) Temperature dependence of the bilayer resistance. At 7.1 K, the sharp transition was observed as in the inset. (b) Schematic illustrations for the current flowing condition above 7.1 K (top) and below 7.1 K (bottom).

**Figure 3:**
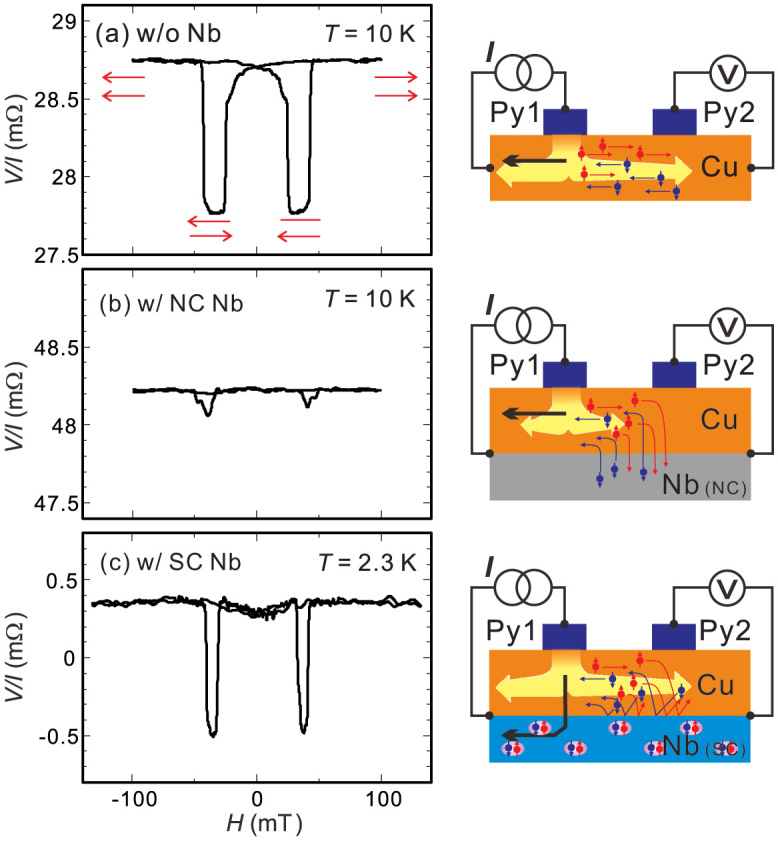
(a) Nonlocal spin valve curve measured at 10 K in the nanopillar based Py/Cu lateral spin valve without the Nb layer together with the probe configuration for the measurement (right). Nonlocal spin valve curves measured at 10 K (NC state) (b) and 2.3 K (SC state) (c) together with the respective probe configurations.

**Figure 4:**
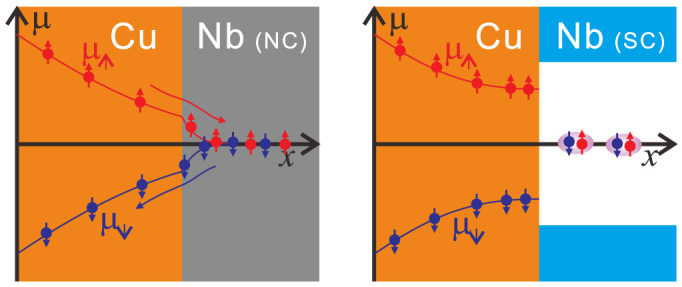
Schematic illustrations of the spatial distribution of the electro-chemical potential at the Nb/Cu interface. The NC Nb strongly absorbs the spin current, resulting in the large reduction of the spin accumulation in the Cu. The SC Nb cannot absorbs the spin current because of the superconducting gap.

**Figure 5:**
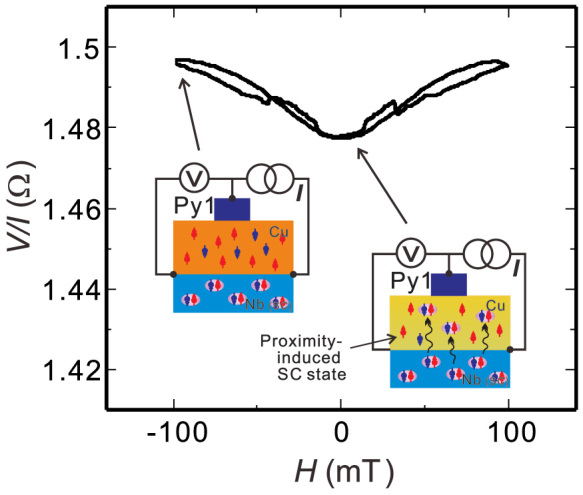
Magnetic field dependence of the contact resistance of the Py1/Cu junction together with the probe configuration for this measurement. The insets show the schematic illustration of the conducting state in the Cu/Nb layer.
